# Supplementary figures and images for: Amyloid precursor protein modulates cerebellar Purkinje cell activity and motor function through regulation of Nav1.6 currents
Source: PLoS Biol. 2025 Nov 24;23(11):e3003513. doi: 10.1371/journal.pbio.3003513 (PMC12668622; doi:10.1371/journal.pbio.3003513)

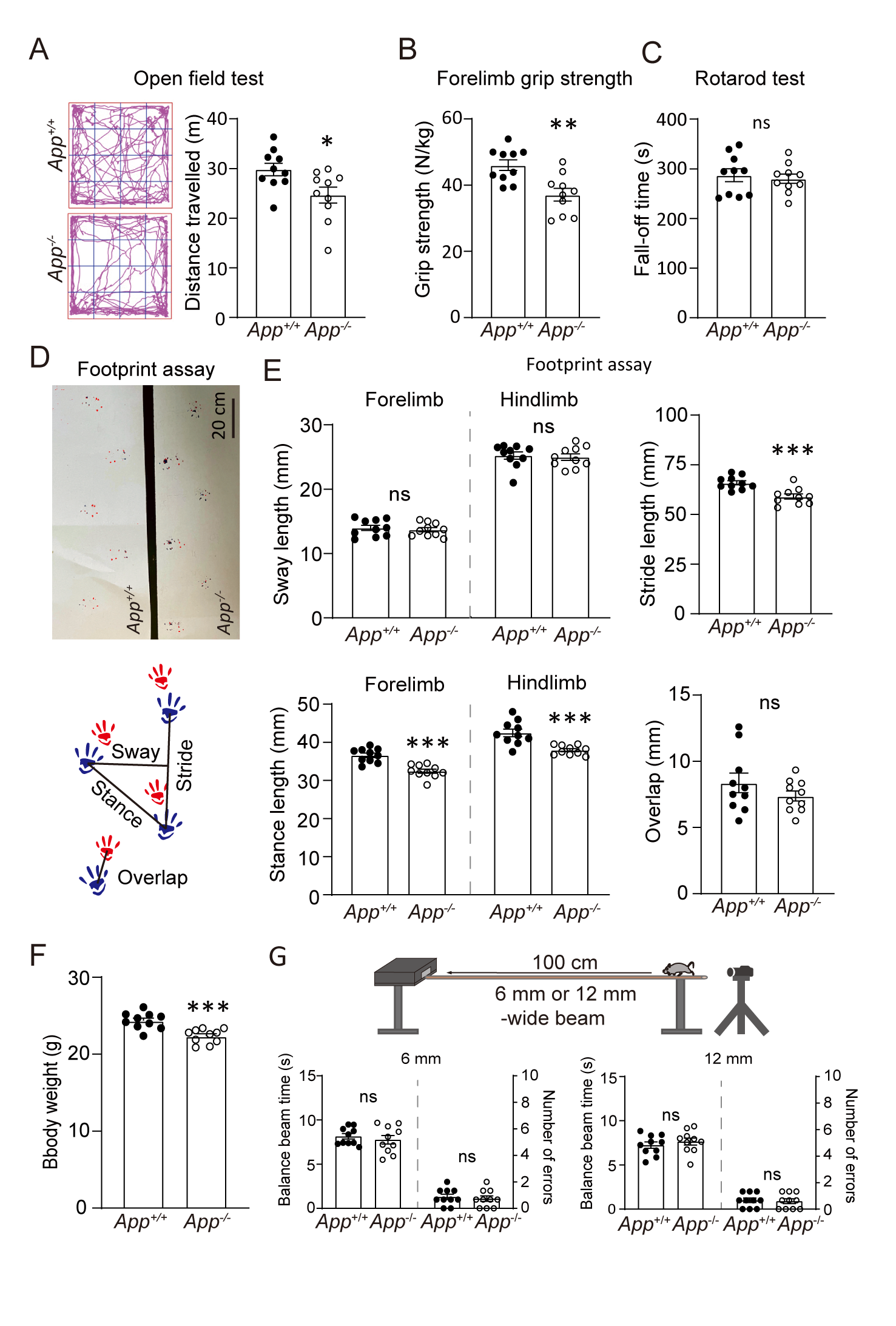

Supplement: S1 Fig — A battery of motor function tests was performed in App+/+ (wild-type) and App−/− mice, n = 10 for each group. (A) Open field test: representative trajectory plots and statistics of the distance traveled in the open field. (B) Statistics of grip strength test. (C) Statistics of rotarod test. (D) Representative trajectory maps and schematic measurement of footprint. (E) Statistics of sway length, stride length, stance length and overlap of the footprint assay in App+/+ and App−/− mice. (F) Statistics of mouse body weight. (G) Balance beam test. The time taken for mice to traverse beams of two different widths (6 and 12 mm) and the number of slips were recorded. Bar-charts show the quantification of traversal time and number of slips per mouse. N = 10 for each group. Scale bars are indicated in the images. Student t test: * P < 0.05; ** P < 0.01; *** P < 0.001; ns, non-significant. The data underlying this Figure can be found in S1 Data. (TIF) [file pbio.3003513.s001.tif]

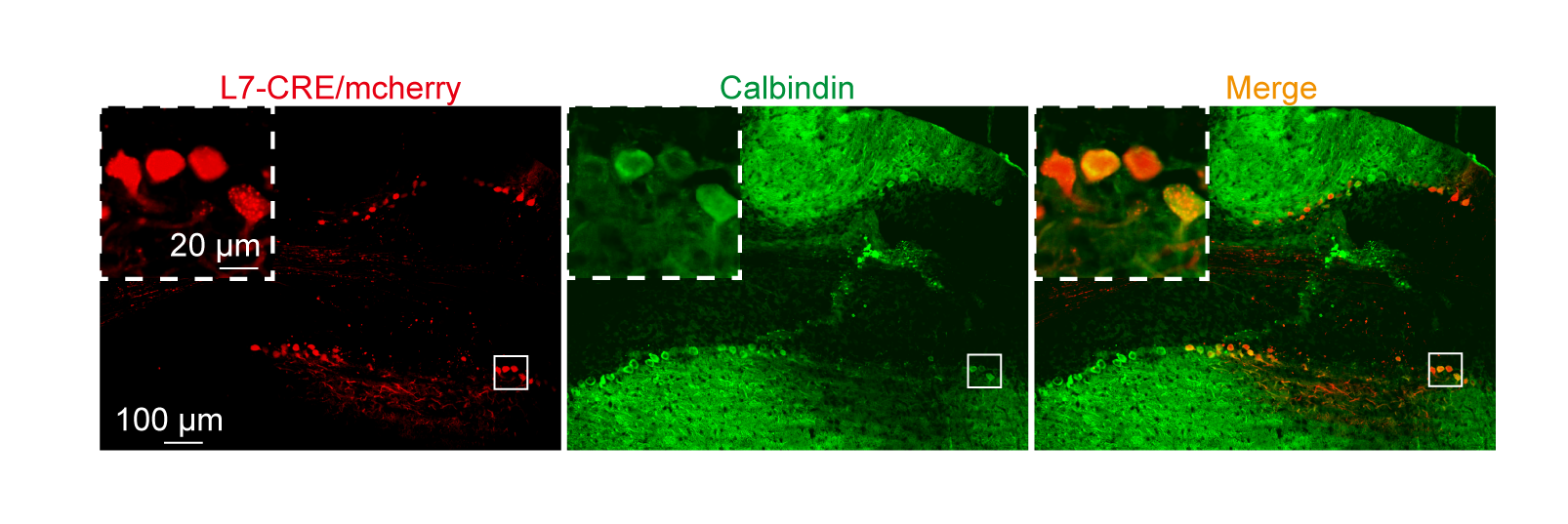

Supplement: S2 Fig — Representative sagittal cerebellar sections (20 μm) from wild-type mice injected with rAAV-L7-CRE-P2A-mCherry-WPRE-hGH, showing mCherry (Cre reporter, red) and immunofluorescence for the PC marker calbindin (green). Merged images reveal exclusive co-localization within PCs, confirming recombinant expression specificity. Scale bars: 100 μm (main); 20 μm (insets). (TIF) [file pbio.3003513.s002.tif]

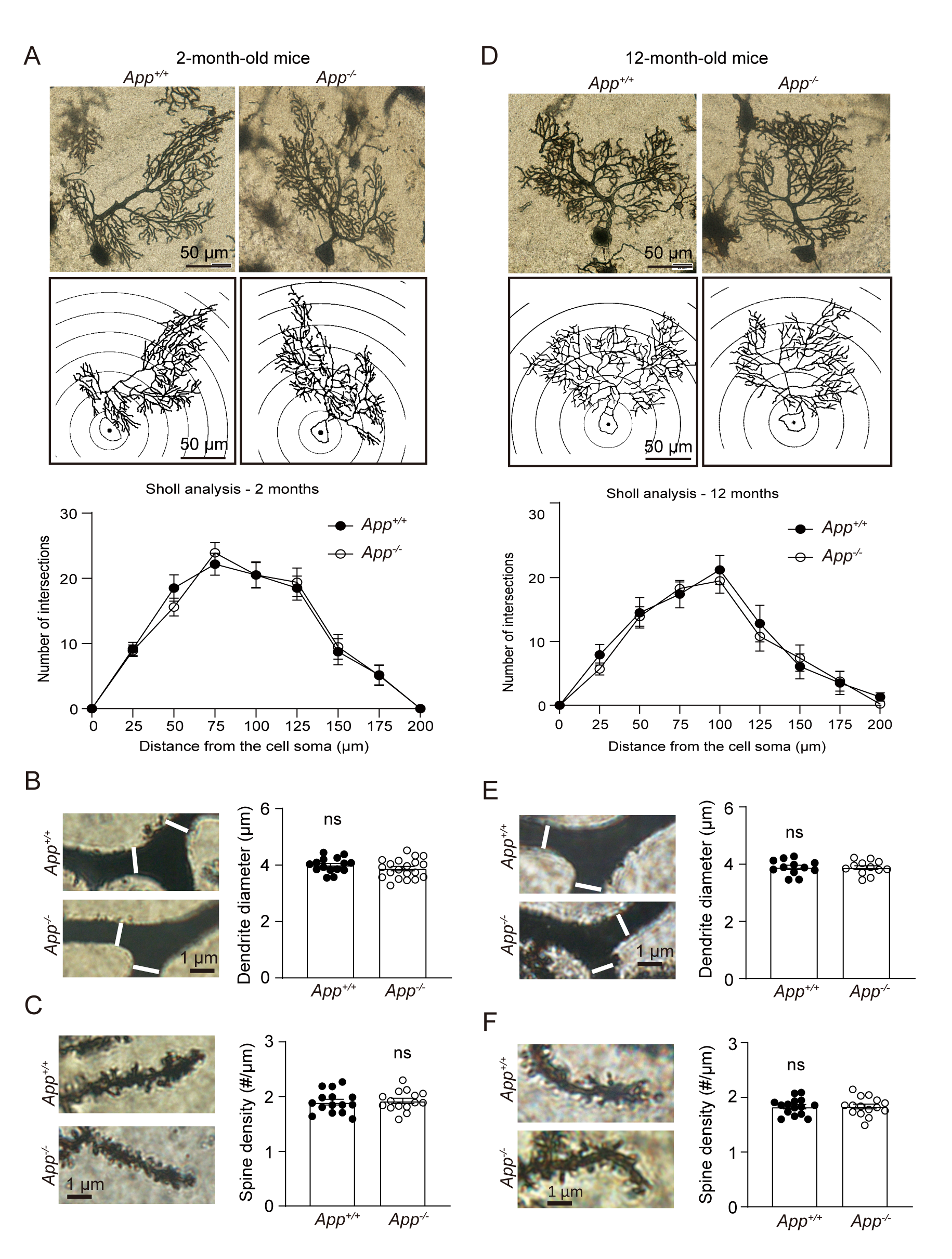

Supplement: S3 Fig — (A–C) Analyses in 2-month-old and (D–F) 12-month-old App−/− versus App+/+ mice. (A, D) Representative sagittal sections of the cerebellar cortex showing Golgi-stained PCs, with corresponding Sholl analysis of dendritic arborization. Concentric circles (radius increment: 25 µm) are overlaid on the binary-traced PC image to quantify intersections with the dendritic processes. App+/+ (2-month-old), n = 13; App−/− (2-month-old), n = 12; App+/+ (12-month-old), n = 10; App−/− (12-month-old), n = 10. (B, E) Measurement of primary dendrite diameter at a distance of 10 μm from the soma. App+/+ (2-month-old), n = 15; App−/− (2-month-old), n = 19; App+/+ (12-month-old), n = 12; App−/− (12-month-old), n = 12. (C, F) Quantification of dendritic spine density on distal dendrites of PCs. Red arrowheads indicate spines along a distal dendrite. App+/+ (2-month-old), n = 14; App−/− (2-month-old), n = 14; App+/+ (12-month-old), n = 15; App−/− (12-month-old), n = 15. Scale bars are indicated in the images. Data are from 4 mice per group; Student t test, ns: not significant. The data underlying this Figure can be found in S1 Data. (TIF) [file pbio.3003513.s003.tif]

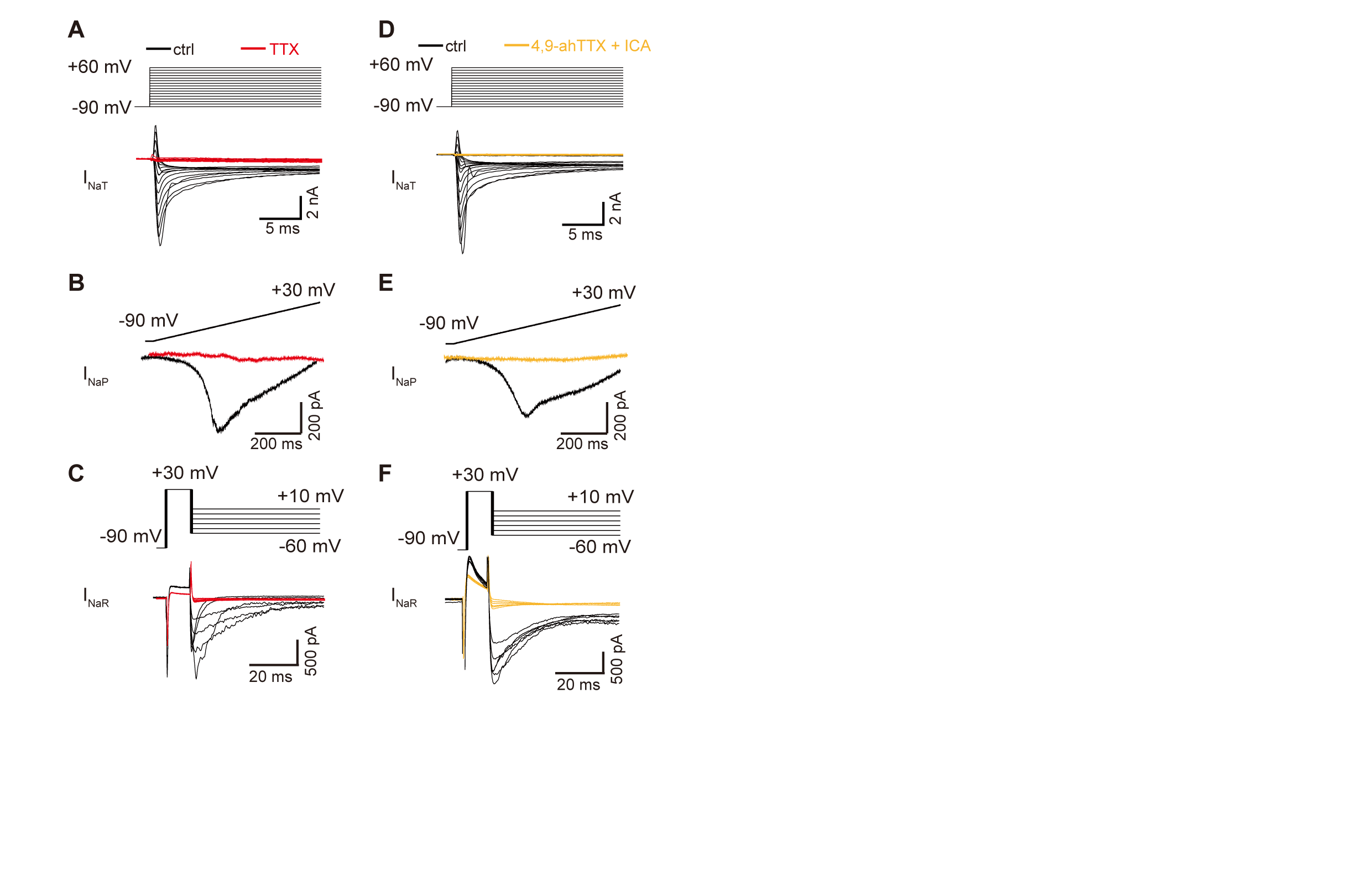

Supplement: S4 Fig — Whole-cell voltage-clamp recordings from wild-type (App+/+) PCs characterizing voltage-gated sodium current subtypes. (A, D) Transient current (INaT): elicited by depolarizing steps from −90 to +60 mV. (B, E) Persistent current (INaP): evoked using a 1,000-ms voltage ramp from −90 to +30 mV. (C, F) Resurgent current (INaR): induced following a +30 mV step (from Vhold = –90 mV) with repolarizing steps from −60 to +10 mV (10 mV increments). (A–C) Current traces before (black) and during application of 1 µM tetrodotoxin (TTX, pan-NaV blocker; red). (D–F) Current traces before (black) and during co-application (orange) of 200 nM 4,9-anhydrotetrodotoxin (Nav1.6 blocker) and 350 nM ICA-121431 (Nav1.1 inhibitor). Scale bars are shown in the figure. (TIF) [file pbio.3003513.s004.tif]

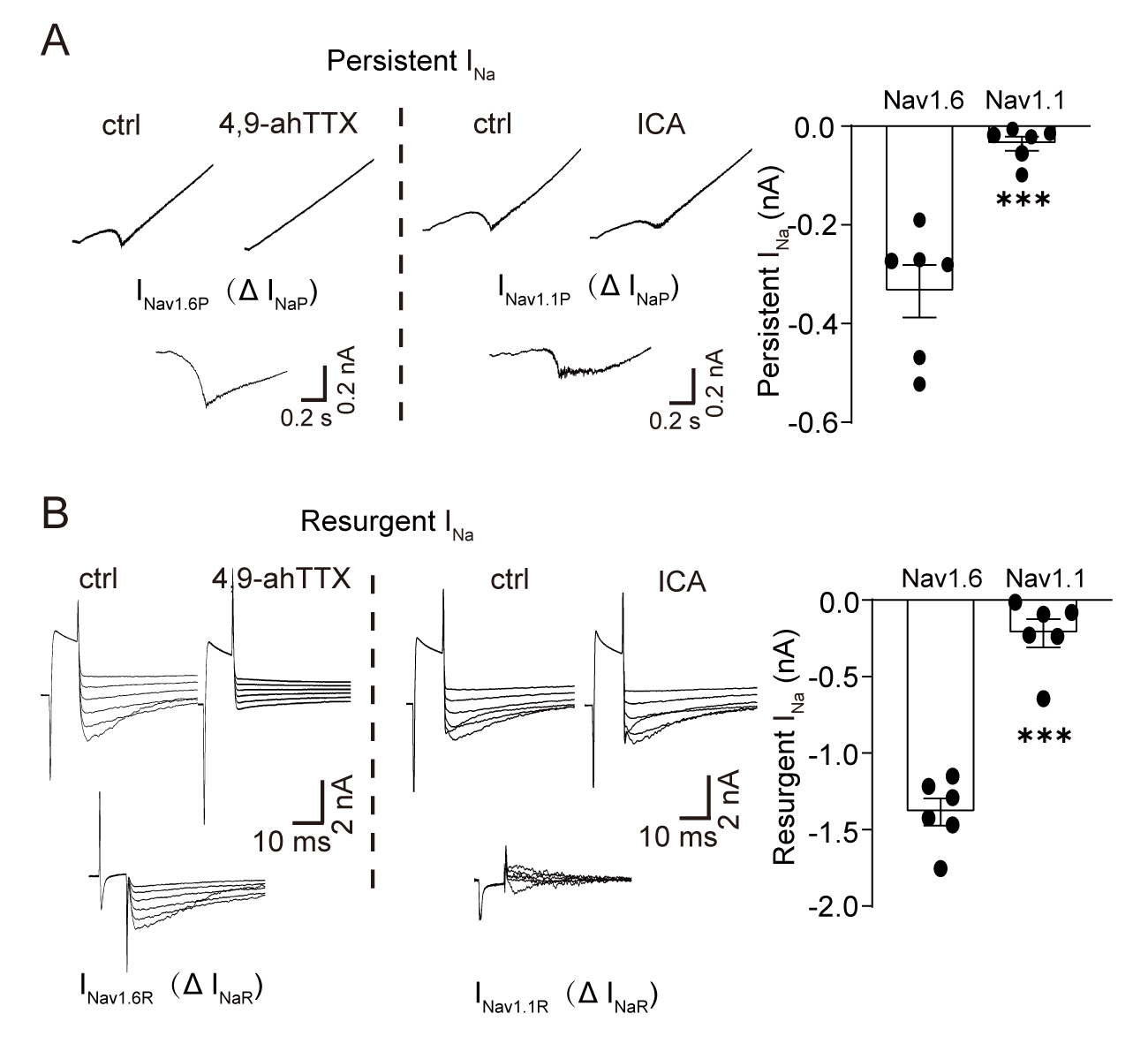

Supplement: S5 Fig — (A) Persistent Na⁺ currents elicited by a 1,000-ms ramp from −90 to +30 mV. Representative traces and statistical bar-charts showing amplitude of persistent INa before or during bath application of Nav1.6 specific blocker 4,9-ahTTX (200 nM), or Nav1.1 specific inhibitor ICA-121431 (ICA, 350 nM) in wild-type (App+/+) mice. 4,9-ahTTX: n = 6 cells, ICA: n = 6 cells. (B) Resurgent Na⁺ currents elicited following a step to +30 mV (from a holding potential of −90 mV) by a series of 10 mV depolarizing voltage steps from −60 to +10 mV. Representative traces and statistical bar-charts showing amplitude of resurgent INa before or during bath application of 4,9-ahTTX or ICA in wild-type mice. 4,9-ahTTX: n = 6 cells, ICA: n = 6 cells. The Nav1.6 or Nav1.1 currents were determined by subtracting the sodium currents recorded during bath application of specific blockers/inhibitors from that before drug application. Scale bars are indicated in the figure. Statistics: Unpaired t test; *** P < 0.001. The data underlying this Figure can be found in S1 Data. (TIF) [file pbio.3003513.s005.tif]

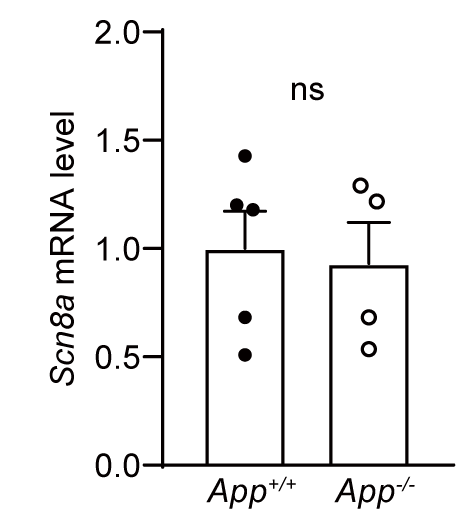

Supplement: S6 Fig — QPCR analysis of LCMed cerebellar PCs in App+/+ or App−/− mice. Statistics: App+/+, n = 5; App−/−, n = 4. Unpaired t test; ns, non-significant. The data underlying this Figure can be found in S1 Data. (TIF) [file pbio.3003513.s006.tif]

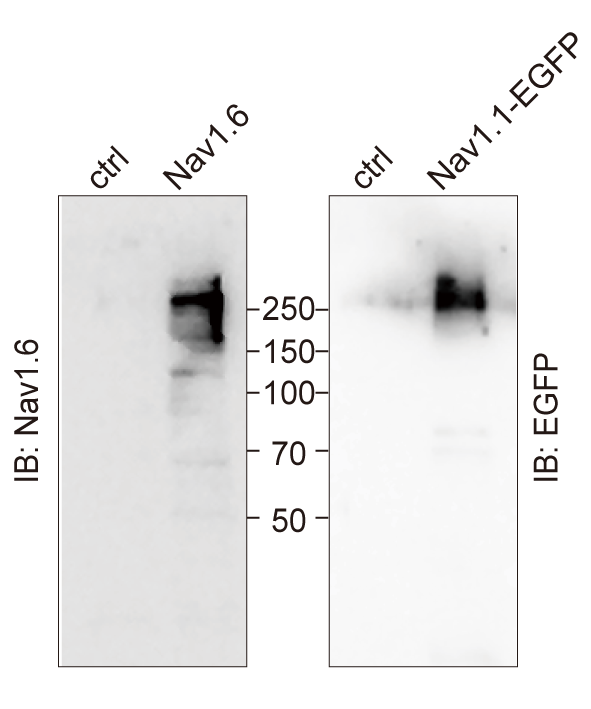

Supplement: S7 Fig — Total lysates of HEK293 cells expressing either Nav1.6 or Nav1.1-GFP were immunoblotted with Nav1.6 (Alomone Labs #ASC-009) or EGFP (Roche Applied Science, #11814460001) antibody. (TIF) [file pbio.3003513.s007.tif]

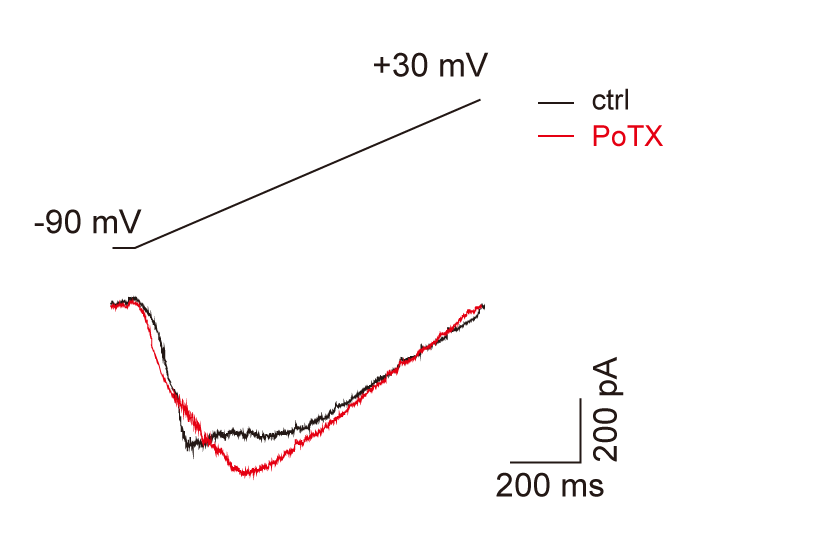

Supplement: S8 Fig — Whole-cell voltage-clamp recordings of persistent sodium currents in App−/− PCs before (black) and during bath application of 30 nM PoTX (red). Currents were elicited by a 1000-ms ramp to +30 mV from a holding potential of −90 mV. Scale bars as indicated. (TIF) [file pbio.3003513.s008.tif]

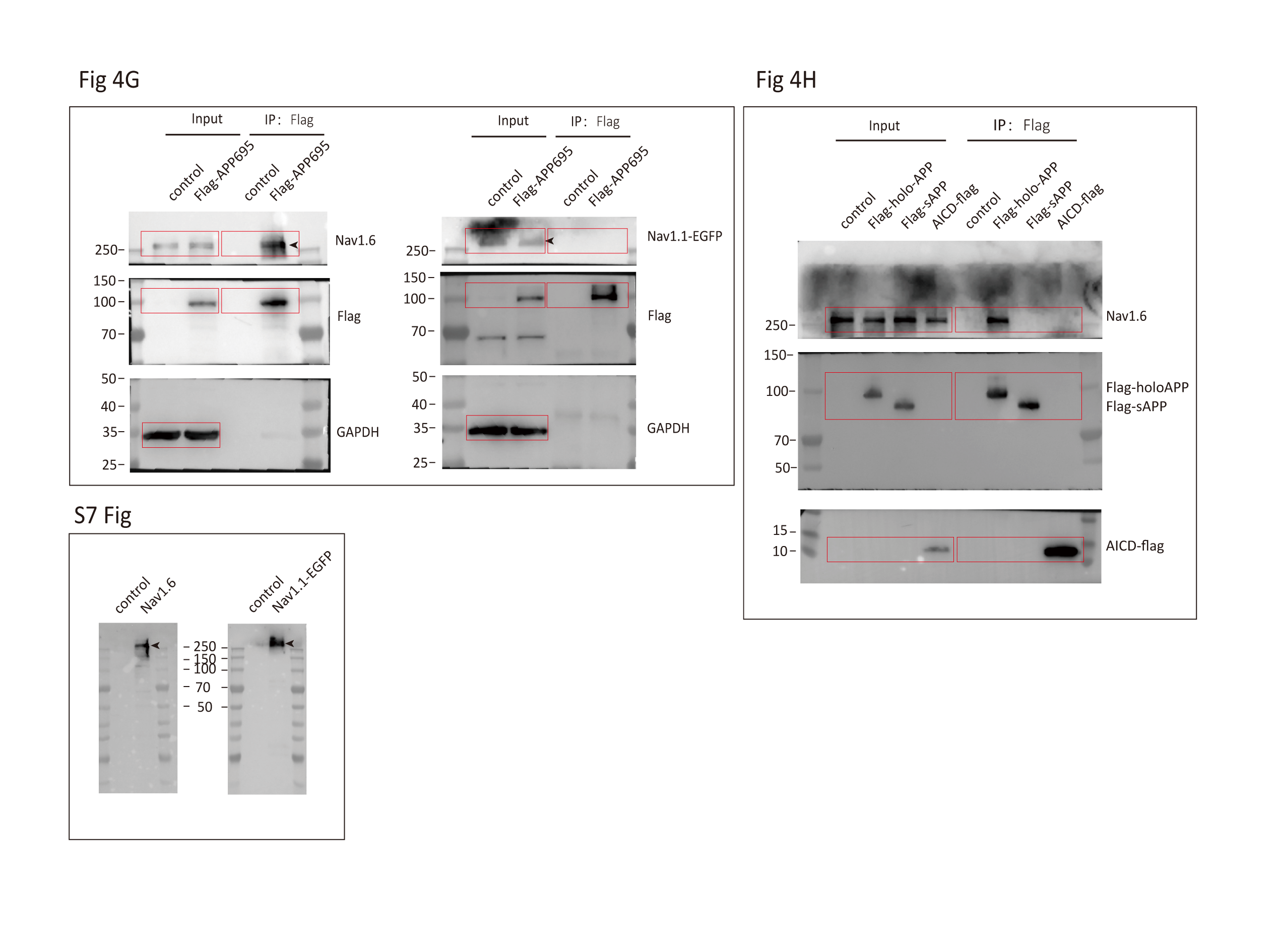

Supplement: S1 Raw Images — (TIF) [file pbio.3003513.s009.tif]
