# Supplementary material for: Amyloid precursor protein modulates cerebellar Purkinje cell activity and motor function through regulation of Nav1.6 currents
Source: PLoS Biol. 2025 Nov 24;23(11):e3003513. doi: 10.1371/journal.pbio.3003513 (PMC12668622; doi:10.1371/journal.pbio.3003513)
Supplement: Attachment — Submitted filename: PB rebuttal letter.docx [file pbio.3003513.s011.docx]

**Rebuttal Letter**

REVIEWS:

Reviewer #1: The manuscript by Ji et al. presents a well-designed study with novel insights into the role of APP in cerebellar motor control. Mechanistically, the authors demonstrated that APP regulates the firing pattern of PCs and inhibitory synaptic transmission from PCs to DCN by modulating Nav1.6 currents, ultimately influencing the motor function. Exogenous expression of APP in Purkinje cells rescues motor-deficits in APP KO mice. In general, the study reveals a novel physiological role for APP and provides new insights into the molecular mechanisms underlying this process. However, I have several concerns and suggestions outlined below.

Major Comments:

1. The present data demonstrated that APP-null mice exhibited reduced locomotor activity and weaker forelimb grip strength compared to WT littermates, and exogenous APP expression in PCs rescued motor deficits in APP-null mice. However, to confirm the essential role of cerebellar APP in motor regulation, it is beeper to perform conditional knockout of APP specifically in PCs.

**Response: We thank the reviewers for this constructive suggestion. To definitively establish cerebellar APP’s role in motor regulation, we performed conditional APP knockdown in PCs using cerebellar injections of AAV-L7-Cre-IRES-mcherry + AAV-CMV-DIO-(EGFP-U6)-shRNA(APP), with AAV-CMV-DIO-(EGFP-U6)-shRNA(scrambled) as control (Fig 1G).** **The L7 promoter showed exclusive PC-specificity (colocalization with calbindin, new S2 Fig). LCMed PCs exhibited >50% APP mRNA reduction (Fig 1H). Crucially, this PC-specific APP knockdown recapitulated the motor deficits (reduced locomotion, impaired grip strength) observed in full APP-null mice (Fig 1I-J), confirming PC APP’s indispensable role in motor coordination. These results were described in Line 136-145.**

**S2 Fig**

**Fig 1**

2. The authors attribute footprint test differences to body size but do not present body weight data. Including body weight comparisons between WT and APP-null mice would strengthen this claim. Moreover, please explain why the rotarod test shows no effects in APP KO or APP KO+APP.

**Response: We thank the reviewer for this constructive feedback. We have now incorporated body weight measurements for all cohorts in revised S1F Fig. Consistent with prior reports (Zheng et al., *CELL*, 1995, PMID: 7758106), APP-null mice exhibited significantly reduced body mass (WT: 24.4 ± 0.3 g vs. APP-null: 22.4 ± 0.9 g; ***, p < 0.001), which directly explains the altered stride parameters in footprint analyses. we have also conducted the beam walking assay to rigorously evaluate motor coordination (S1G Fig). Our results demonstrate no significant difference in performance between WT and APP-null mice (p > 0.05). These new data have been integrated into the Results section (Line 94-99 with corresponding methodological details in the Materials and Methods (Line 609-615).**

**The rotarod test assesses integrated motor performance, which is influenced by multiple factors including motor coordination, balance, motor learning, and cardiopulmonary endurance (PMID: 18428540, 20359499). In our study, APP-null mice did not exhibit significant deficits in the rotarod task compared to WT controls. This result is consistent with their normal performance in other motor coordination‐specific assays, such as the gait test and the narrow beam walking test. However, APP-null mice did show clear impairments in tasks requiring sustained locomotion and grip strength, which rely more heavily on muscle tone and neuromuscular function. These findings suggest that APP is not critically involved in motor coordination per se, but rather modulates motor output through mechanisms related to muscle tone or locomotor drive—potentially via its regulation of Nav1.6 currents in Purkinje cells and possibly spinal circuits. Thus, the absence of a rotarod phenotype in APP KO mice aligns with the specific rather than general role of APP in motor control, affecting certain motor domains while sparing others.**

**S1 Fig**

3. In addition to electrophysiological recordings, it is recommended that the authors should examine whether knockout of APP had any effect on the expression level of Nav1.6 in the cerebellum.

**Response: We have examined Nav1.6 (*Scn8a*) expression in cerebellar PCs and detected no significant alterations following APP knockout (S6 Fig). This suggests that APP-mediated regulation of Nav1.6 may occur primarily through modulation of surface channel trafficking, consistent with our previous findings in HEK293 cells (Liu et al., JBC, 2015). This result was described in Line 253-254.**

**S6 Fig**

4. It is necessary to ask whether Nav1.6 inhibitors/blockers could mimic APP KO, while overexpression of Nav 1.6 (or channel activators/enhancers) could rescue the motor deficits in the APP KO mice.

**Response: We addressed this through cerebellar cortical infusion experiments: (1) The Nav1.6 blocker 4,9-anhydrotetrodotoxin (4,9-ahTTX) reduced spontaneous PC firing (Fig 5A) and recapitulated APP-null motor deficits (Fig 5E-G); (2) Conversely, the positive allosteric modulator Poneratoxin enhanced Purkinje cell firing in APP-KO mice (Fig 5B) and rescued motor coordination deficits in behavior tests (Fig 5E-G). These results were described in Line 263-293.**

**Fig 5**

5. While the study focuses on cerebellar PCs, APP is also expressed in other motor-related regions (e.g., spinal cord). The authors should acknowledge that APP in other neuronal populations might contribute to motor deficits, even if PCs are the primary locus (Page 19, last paragraph).

**Response: We thank the reviewer for highlighting this valuable perspective. As suggested, we have now explicitly acknowledged in the Discussion (Line 404-410) that: " However, it should be noted that this study focuses solely on cerebellar PCs; we cannot exclude the possibility that APP also modulates motor function via other regions of the cerebrospinal circuit, such as spinal motor neurons. Nav1.6-mediated persistent sodium current (I_NaP_) contributes to postural tone and amplifies locomotor outputs in spinal cord bistable motor neurons [34]. Given that APP is also expressed in spinal motor neurons [35], it is likely that spinal APP may similarly participate in the regulation of motor function." This addition contextualizes our findings within broader neural circuitry mechanisms.**

6. Critical figures lack sufficient resolution or annotations. For example: (1) Include merged image of APP/Calbindin to confirm the colocalization in Figure 1A. (2) Enhance the resolution of Golgi staining images and label dendritic spines/intersections clearly with arrows (or arrowheads) in Supplemental Figure 2. (3) Ensure that all the scale bars, group labels, and statistical annotations (e.g., asterisks) are legible.

**Response: We have now: 1) Added a merged APP/Calbindin image with colocalization and used APP-null mice as a control in Fig 1A; 2) presented high-resolution Golgi staining images in S3 Fig, using concentric circles to denote dendritic intersections and red arrowheads to highlight dendritic spines. 3) Ensured all scale bars, group labels, and statistical annotations (including asterisks) are prominently displayed at publication-quality resolution throughout the manuscript.**

**Fig 1**

**S3 Fig**

Minor Comments:

1. In Page 15, replace "mediated by the Nav1.1-Nav1.6 partnership in PCs" with "mediated by Nav1.1 and Nav1.6 in PCs" for clarity.

**Response: we have replaced the wording as suggested in Line 219-220: “Voltage steps (-70 mV to +10 mV) from a holding potential of -90 mV elicited I_NaT_, mediated by Nav1.6 and Nav1.1 (TTX/4,9-ahTTX + ICA-sensitive, S4A, S4D Fig)”.**

2. Define all abbreviations upon first use to improve readability for non-specialists. For instance, “4,9-ahTTX” should be spelled out as “4,9-anhydrotetrodotoxin”.

**Response: We have defined all abbreviations throughout the manuscript at first use.**

3. Explicitly state that the study focuses solely on cerebellar PCs and does not explore APP’s role in other motor pathways (e.g., corticospinal tracts). This limitation should be addressed in the Discussion.

**Response: As suggested, we have now explicitly stated in the Discussion (Line 404-410) that: " However, it should be noted that this study focuses solely on cerebellar PCs; we cannot exclude the possibility that APP also modulates motor function via other regions of the cerebrospinal circuit, such as spinal motor neurons. Nav1.6-mediated persistent sodium current (I_NaP_) contributes to postural tone and amplifies locomotor outputs in spinal cord bistable motor neurons [34]. Given that APP is also expressed in spinal motor neurons [35], it is likely that spinal APP may similarly participate in the regulation of motor function."**

4. Fig. 2 and Fig. 5. AP rheobase should also be included into the analysis.

**Response: We have added the rheobase analysis in revised Fig 3C & Fig 6G, and described in Line191-192 & Line 328-329.**

**Fig3**

**Fig 6**

5. Fig. 3D should have a title for the horizontal axis.

**Response: We have added "Membrane potential (mV)" for the horizontal axis in Fig 3D.**

6. Fig. 6B. Scale bar: µm, not uM.

**Response: We have corrected the typographical error in revised Fig 7B.**

Reviewer #2: This study provides an elegant demonstration of the importance of APP in motor control and in cerebellar purkinje cell activity, via modulation of the Nav1.6 sodium channels. The study is overall well performed and experiments are generally well controlled. This brings in vivo confirmation of the interplay between APP and Nav1.6 that the authors highlighted in a previous study in heterologous HEK293 cells. Yet, it would have been a plus if the authors had pushed the study further to bring more information on the molecular mechanism at play. For example, it could have been interesting to identify if full length APP is necessary for the rescue of the cell activity or/and behavioral activity phenotypes or peptides derived from its processing are sufficient, such as for example the C-terminal domain (AICD) or the soluble fragment of APP. Along the same line, the authors do not provide any novel information on how APP regulates Nav1.6 activity beyond what they have published previously in HEK293 cells.

**Response: We thank the reviewer for the insightful comments. We have now added critical data demonstrating that full-length APP is required for functional rescue. As shown in revised Fig 2, neither sAPPα nor AICD fragments rescued behavioral deficits in APP-null mice. Furthermore, co-immunoprecipitation assays revealed that full-length APP specifically interacts with Nav1.6 but not Nav1.1 (Fig 4G), explaining its selective regulation of Nav1.6 channel activity. This binding was dependent on holo-APP, as neither the soluble ectodomain sAPPα nor the APP intracellular domain (AICD) co-precipitated with Nav1.6 (Fig 4H), consistent with the requirement of holo-APP for functional rescue observed in vivo (Fig 2). These findings provide novel mechanistic evidence beyond our prior HEK293 work by establishing: 1) the necessity of full-length APP for in-vivo function, and 2) isoform-specific binding underlying channel regulation. We have described these new results in Line 149-172 & Line 255-258.**

**Fig 2**

**Fig 4**

Beyond this first observation, I have some major and minor comments regarding the presented work that should be addressed:

1. The example of APP staining in Figure 1A does not show strong expression of APP in all the cell bodies that are then highlighted with arrows. Could the authors provide better images or a quantification to show APP levels in calbindin positive neurons (using APP null mice as controls)?

**Response: We have provided better images demonstrating APP expression in calbindin-positive neurons in the revised Fig 1A. These now include the APP-null mice as negative controls.**

**Fig 1**

2. In their manuscript, the authors explain the decrease in spontaneous firing rate of PC of APP null mice by modifications in sodium Nav 1.6 channel function. However, in Figure 2, together with this decreased firing rate, they observe higher threshold for AP generation and higher AHP. To my opinion, these modifications could also explain the decrease in firing rate. They authors do not address this point. The authors should at least acknowledge this possibility as additional modifications brought about by loss of APP and discuss this point. Please also add 'spontaneous' firing rate because they are not inducing this firing with depolarization steps.

**Response: We acknowledge the reviewer's valuable insight that elevated action potential threshold and enhanced afterhyperpolarization (AHP) likely contribute to reduced firing frequency in APP null mice. We have expanded the Discussion section (Line 436-440) to explicitly address these factors as complementary mechanisms alongside Nav1.6 alterations: “However, as shown in Fig 2, APP-null mice exhibited a higher threshold for** **action potential generation and an enhanced AHP. These electrophysiological alterations may collectively contribute to the observed reduction in spontaneous firing frequency of PCs. In addition to regulating Nav1.6, APP may also modulate the threshold for AP generation and AHP through other mechanisms”.**

**Additionally, we have systematically revised all instances of "firing rate" to "spontaneous firing rate" throughout the manuscript to clarify the unstimulated recording conditions.**

3. Figure 2B: authors describe three profiles of PC activity in WT. Is this expected? If so, they should cite previous work evidencing these different firing patterns.

**Response: Yes, the observed heterogeneity in PC firing patterns is expected and aligns with established electrophysiological characteristics of cerebellar neurons. As previously documented (PMID 9354334, 29741614, 29982917), PCs display three characteristic firing states: silent, simple spikes, and complex spikes. We have now explicitly cited this foundational study in the revised manuscript (Line 182-184) to contextualize our observations within existing mechanistic frameworks.**

4. In Figure 3, the authors provide recordings of what they assume are transient, persistent and resurgent sodium currents. To ensure that they are indeed only measuring these currents, they should provide additional data. For example, they should show that these currents are gone in presence of sodium channel blockers.

**Response: We thank the reviewer for this essential methodological suggestion. New data in S4 Fig now demonstrate almost complete abolition of recorded currents following:**

**Application of 1 μM tetrodotoxin (TTX), confirming their sodium channel dependence;**

**or selective pharmacological inhibition of Nav1.6 (200 nM 4,9-ahTTX) and Nav1.1 (350 nM ICA).**

**These controls validate that the observed transient, persistent, and resurgent currents are mediated almost exclusively by Nav1.6 and Nav1.1 channels in PCs, with no significant contribution from other voltage-gated sodium channel isoforms.**

**S4 Fig**

5. Figure 4 A-D is difficult to understand. In A and C, authors refer to the channel (Nav1.6 or Nav 1.1) in the x-axis, but in C-D authors refer to the drug being used. Also, in C-D, they demonstrate effect in presence of 4,9-ahTTX or ICA by substracting the control currents. What do they mean by this as I don't really understand the nA values reported in C (far right) and D (far right) after this substraction. Would it not be more appropriate to normalize the data in C or D to the average current obtained in ACSF (as 100%) to remove this variable in peak current observed in ACSF WT and APP KO to then correctly assess the drug effect?

**Response: We sincerely apologize for the lack of clarity in the original presentation. The figure has been comprehensively restructured:**

**Original panels in Figure 4A-B (demonstrating Nav1.6 dominance in persistent/resurgent currents) are now in S5 Fig.**

**Panels in Figure 4C-D have been reconfigured as new Fig 4C-D to explicitly show APP-deficiency effects on isolated Nav1.6/Nav1.1 currents.**

**Regarding methodological concerns (subtraction protocol): The "Control - Drug" subtraction isolates channel-specific currents (Fig 4C, D), not showing drug effects.**

**Fig 4**

6. Last sentence of sub-chapter 4 of Results: the authors write 'These data provide evidence that APP regulates PC firing through Nav1.6'. Where are the data in APP null mice for E-F? How can the authors conclude this from this experiment in E-F?

**Response: We thank the reviewer for this important critique. New data now demonstrate that: (1) Application of the Nav1.6-specific positive modulator poneratoxin to APP-null PCs rescues firing deficits (Fig 5B), while (2) Nav1.6 blockade (4,9-ahTTX) in WT cells recapitulates the APP-null phenotype in PCs (Fig 5A). These gain- and loss-of-function experiments provide mechanistic evidence supporting our conclusion that "APP regulates PC firing through Nav1.6". We have described these results in Line 264-268.**

**Fig 5**

7. Writing all the results details (numbers, s.e.m., statistics, etc….) within the main text makes the Results section very difficult to read. I would suggest to provide supplementary tables with all these details and only describe the main information for each result obtained with maybe add brief statistics information (e.g. type of test used) in the legend for each Figure panel with statistics.

**Response: We thank the reviewer for suggestion. We now only describe the main information for the results. the brief statistics are described in the figure legends, and details in S1 data file.**

Minor:

1. Authors should harmonize how they want to call the model: APP null, APP-null or APP KO?

**Response: We thank the reviewer for highlighting this inconsistency. The terminology has been standardized to "APP-null" throughout the revised manuscript as recommended.**

2. Authors should check for punctuation and spaces when providing the reference citations in the text.

**Response: We have thoroughly revised all in-text citations to ensure consistent punctuation and spacing in accordance with journal style guidelines.**

3. First paragraph of sub-chapter 2 in Results: authors state 'These results indicated that APP deficiency does not affect the development or degeneration of PCs'. To my opinion, this sentence might be mis-interpreted here. I assume that the authors mean is that, in absence of APP, the morphology of PC cells in normal even in old mice. If so, they should write 'nor provoke degeneration of PCs'.

**Response: We thank the reviewer for highlighting this important nuance. The sentence has been revised to: "These results indicate that APP deficiency affects neither Purkinje cell development nor provokes their degeneration" in Line 180-181 to precisely convey the absence of pathological effects on cellular integrity across ages.**

4. Title of sub-chapter 2 of Results: the title does not make sense - please correct the syntax.

**Response: We have restructured this heading to: "APP deficiency impairs PC electrophysiological activity." in Line 174 to accurately reflect the functional deficits documented in this section.**

5. Figure 2B: modify red label to read 'silent' not 'slient'.

**Response: We thank the reviewer for identifying this error. The label in Fig 2B has been corrected to "silent" in the revised Fig 3A.**

Reviewer #3: The manuscript by Ji et al. explores the role of Amyloid precursor protein (APP) in motor behavior and cerebellar physiology. The authors demonstrated that motor impairments, such as deficits in grip strength and locomotion observed in APP-null mice, can be rescued by APP gene expression in Purkinje cells (PC). At the cellular level, the authors investigate the impact of APP on PC activity using patch-clamp analysis, with a particular focus on the Nav1.6 and Nav1.1 voltage-gated sodium channels. The manuscript is well-written and offers insights into the involvement of the cerebellum in the pathogenesis of Alzheimer's disease.
Major revisions:

1. In Figure 1 I and J, no significant difference between WT and KO mice is shown. Why did the authors present similar data in Suppl. Figure 1? The only difference between the two sets of figures is the virus injections. Since the main message is already conveyed in Figure 1, the supplementary figure appears redundant and unnecessary.

**Response: We thank the reviewer for this insightful observation. We have now reorganized Figure 1 and S1 Fig to eliminate redundancy and enhance conceptual clarity. The revised S1 Fig serves specifically to document baseline motor function screening across genotypes, while Fig 1 focuses exclusively on demonstrating functional rescue of the behavioral deficits identified in this screening. This separation ensures each figure maintains distinct experimental objectives: establishing baseline phenotypes (S1 Fig) versus testing mechanistic rescue (Fig 1).**

2. I recommend testing the mice using the beam walking assay to more accurately assess motor coordination and support the conclusion that "the aberrant gait pattern of APP-null mice was due to the body size instead of APP deficiency".

**Response: We sincerely thank the reviewer for this valuable suggestion. In response, we have conducted the beam walking assay to rigorously evaluate motor coordination. Our results demonstrate no significant difference in performance between WT and APP-null mice (p > 0.05) (S1G Fig). Furthermore, we have incorporated body weight measurements in S1 Fig (S1F Fig) to provide additional evidence supporting our conclusion that the observed gait alterations in APP-null mice are attributable to body size differences rather than APP deficiency. These new data have been integrated into the Results section (Line 94-97) with corresponding methodological details in the Materials and Methods (Line 609-615).**

**S1 Fig**

3. Both the results and the figures related to the Nav analysis require improvement. For example, in Figure 4, I recommend displaying the current traces before and after the application of the inhibitors, indicated above the traces. The authors should also include a figure showing the effects of the simultaneous application of Nav1.6 and Nav1.1 inhibitors to better demonstrate the contribution of these channels to the PC Na currents being recorded.

**Response: We sincerely thank the reviewer for these constructive suggestions. In response, we have now: 1) displayed representative current traces before and after application of specific blockers/inhibitors in revised Fig 4C-D (transient I_Na_), S5A Fig (persistent INa), and S5B Fig (resurgent INa), with clear drug application indicated above each trace; and 2) included new data demonstrating the combined effects of Nav1.6 and Nav1.1 inhibitors in S4D-4F Fig. These controls validate that the observed transient, persistent, and resurgent currents are mediated almost exclusively by Nav1.6 and Nav1.1 channels in PCs, with no significant contribution from other voltage-gated sodium channel isoforms. These comprehensive additions have been fully incorporated into the Results section (Line 219-220), significantly strengthening our analysis of Nav channel contributions to PC sodium currents.**

**Fig 4**

**S4 Fig**

4. Figure 6B needs improvement as the signal appears highly dispersed.

**Response: We appreciate the reviewer's careful evaluation of Figure 6B. In response to this concern, we have: 1) repeated the imaging experiments with optimized acquisition parameters, 2) replaced the original panels with more clear fluorescence images. The revised Fig 7B now provides improved visualization of the signal localization.**

**Fig 7**

Minor revisions

1. Since the APP-null mice show impairments in the grip test, the statement "these results support the conclusion that APP-null mice exhibit normal neuromuscular functions" is contradictory. Please revise or remove this sentence at the end of the first paragraph of the result section.

**Response: Thanks for the suggestion. We have removed the sentence.**

2. In Figures 4E and F, clearly indicate on the top of the traces and within the graphs the type of mice being examined.

**Response: Thanks for the suggestion. we have now clearly indicated the corresponding genotypes above all representative electrophysiological traces throughout the figures. This revision ensures improved clarity and allows readers to more easily correlate the recorded signals with the respective experimental conditions.**

3. The sentence "we show that the reduction in Nav1.6 sodium currents is a key factor contributing to the motor deficits observed in APP knockout mice" needs revision. This is an assumption rather than a direct cause-and-effect relationship, as the study presents associative findings rather than mechanistic proof.

**Response: We appreciate the reviewer's thoughtful comment regarding the causal interpretation of our findings. In response, we have now strengthened the mechanistic evidence in Fig 5 through additional rescue experiments demonstrating that selective Nav1.6 currents restoration in PCs ameliorates APP knockout-induced motor deficits. While we acknowledge that correlation does not equal causation, these new functional studies provide direct experimental support for Nav1.6's role in the observed phenotype. We have revised the results in Line 263-293. We would be happy to consider alternative phrasing should the reviewer/editor recommend further nuance.**

4. In the methods sections related to laser capture microdissection, specify which fluorescence-positive cells were analyzed.

**Response: We have specified “Purkinje cells” in Line 528-531: "Following identification of fluorescence-positive PCs based on characteristic morphology (large flask-shaped somata, 20–30 μm diameter), targeted cells were collected via ultraviolet laser pressure catapulting (PALM MicroBeam) using inverted-beam geometry for anti-gravitational ejection into adhesive cap tubes (AdhesiveCap 500, Zeiss)."**

5. In Figure 2B, change the label to silent.

**Response: We thank the reviewer for identifying this error. The label in revised Fig 3A has been corrected to "silent" in the revised manuscript.**
