# Supplementary material for: Amyloid precursor protein modulates cerebellar Purkinje cell activity and motor function through regulation of Nav1.6 currents
Source: PLoS Biol. 2025 Nov 24;23(11):e3003513. doi: 10.1371/journal.pbio.3003513 (PMC12668622; doi:10.1371/journal.pbio.3003513)
Supplement: Attachment — Submitted filename: Response to reviewers.docx [file pbio.3003513.s012.docx]

We sincerely thank the editors and reviewers for their constructive feedback and positive assessment of our revised manuscript. We are pleased that the revisions have addressed the concerns raised. Below, we provide our responses to the specific comments.

Reviewer #1:

My points raised in the previous round of review have been satisfactorily addressed. Now the paper is acceptable for publication.

**Response:**

**We thank Reviewer #1 for their positive feedback and confirmation that our revisions have satisfactorily addressed their previous concerns.**

Reviewer #3:

The revised manuscript shows substantial improvement, and the authors have addressed most of my concerns.

**Response:**

**We are grateful to Reviewer #3 for acknowledging the improvements in the revised manuscript and for their valuable input during the review process.**

Comments from the Academic Editor:

This new version of the manuscript provides the necessary additional information requested. The new experiments, which were carefully designed, significantly enhance the impact of the manuscript.

I only have one minor comment that would require a sentence in the discussion:

In figure 5B, F and G, the authors show that using a positive allosteric modulator of Nav1.6 they could rescue the firing properties of APP-null PCs and motor deficits in APP-null mice. In the Discussion section, the authors should provide their insights on how this rescue might work mechanistically. In previous work, the authors showed that APP regulates Nav1.6 by enhancing its cell surface distribution. How do the authors link these two observations?

I also reviewed the responses to the comments of the two other reviewers. It is my personal feeling that the authors made strong efforts to respond to all of them.

**Response:**

**We are grateful to the Academic Editor for the insightful comments and for acknowledging the work invested in revising our manuscript. Addressing the Editor's minor point, we have incorporated the following statement into the Discussion section to better elucidate the mechanistic connection:**

**“****We further demonstrated that PoTX, a positive allosteric modulator of Nav1.6 that reduces channel inactivation, consequently enhanced the persistent I_Na_—a key determinant of repetitive action potential firing. This PoTX-mediated enhancement likely compensates for the functional deficits resulting from reduced surface distribution of Nav1.6 in APP-null PCs, as evidenced by the restoration of firing properties and amelioration of motor deficits. These results further support the conclusion that Nav1.6 serves as the primary effector mediating APP-dependent regulation of cerebellar PC function.” This addition can be found on Page 15-16 of the revised manuscript.**

Once again, we extend our sincere appreciation to the reviewers and the Academic Editor for their time and insightful comments, which have greatly improved the quality of our work.
